# Health Profiles of Mosaic Versus Non-mosaic *FMR1* Premutation Carrier Mothers of Children With Fragile X Syndrome

**DOI:** 10.3389/fgene.2018.00173

**Published:** 2018-05-16

**Authors:** Marsha R. Mailick, Arezoo Movaghar, Jinkuk Hong, Jan S. Greenberg, Leann S. DaWalt, Lili Zhou, Jonathan Jackson, Paul J. Rathouz, Mei W. Baker, Murray Brilliant, David Page, Elizabeth Berry-Kravis

**Affiliations:** ^1^Waisman Center, University of Wisconsin-Madison, Madison, WI, United States; ^2^Department of Biomedical Engineering, University of Wisconsin-Madison, Madison, WI, United States; ^3^Department of Pediatrics, Rush University Medical Center, Chicago, IL, United States; ^4^Department of Pathology, Rush University Medical Center, Chicago, IL, United States; ^5^Department of Biostatistics and Medical Informatics, University of Wisconsin-Madison, Madison, WI, United States; ^6^Wisconsin State Laboratory of Hygiene, Madison, WI, United States; ^7^Marshfield Clinic Research Institute, Marshfield, WI, United States; ^8^Department of Neurological Sciences and Biochemistry, Rush University Medical Center, Chicago, IL, United States

**Keywords:** *FMR1* premutation, CGG repeats, mosaicism, genotype–phenotype correlations, machine learning

## Abstract

The *FMR1* premutation is of increasing interest to the FXS community, as questions about a primary premutation phenotype warrant research attention. 100 *FMR1* premutation carrier mothers (mean age = 58; 67–138 CGG repeats) of adults with fragile X syndrome were studied with respect to their physical and mental health, motor, and neurocognitive characteristics. We explored the correlates of CGG repeat mosaicism in women with expanded alleles. Mothers provided buccal swabs from which DNA was extracted and the *FMR1* CGG genotyping was performed (Amplidex Kit, Asuragen). Mothers were categorized into three groups: Group 1: premutation non-mosaic (*n* = 45); Group 2: premutation mosaic (*n* = 41), and Group 3: premutation/full mutation mosaic (*n* = 14). Group 2 mothers had at least two populations of cells with different allele sizes in the premutation range besides their major expanded allele. Group 3 mothers had a very small population of cells in the full mutation range (>200 CGGs) in addition to one or multiple populations of cells with different allele sizes in the premutation range. Machine learning (random forest) was used to identify symptoms and conditions that correctly classified mothers with respect to mosaicism; follow-up comparisons were made to characterize the three groups. In categorizing mosaicism, the random forest yielded significantly better classification than random classification, with overall area under the receiver operating characteristic curve (AUROC) of 0.737. Among the most important symptoms and conditions that contributed to the classification were anxiety, menopause symptoms, executive functioning limitations, and difficulty walking several blocks, with the women who had full mutation mosaicism (Group 3) unexpectedly having better health. Although only 14 premutation carrier mothers in the present sample also had a small population of full mutation cells, their profile of comparatively better health, mental health, and executive functioning was unexpected. This preliminary finding should prompt additional research on larger numbers of participants with more extensive phenotyping to confirm the clinical correlates of low-level full mutation mosaicism in premutation carriers and to probe possible mechanisms.

## Introduction

Variation in the number of CGG repeats in the *FMR1* gene has received increasing research attention during the past three decades. Beginning with the discovery in 1991 that fragile X syndrome (FXS) was caused by an expansion of more than 200 CGG repeats in the *FMR1* gene (Verkerk et al., 1991), the importance of this gene for brain development and functioning is now well-established (Darnell et al., 2011). Individuals with FXS have an increased risk of intellectual disability, hyperarousal, anxiety, behavioral dysregulation, and autism spectrum disorder, with symptoms more pronounced in males than in females (Bailey et al., 1998; Abbeduto et al., 2007; Hessl et al., 2008; Smith et al., 2012). FXS is inherited from mothers, almost all of whom carry the *FMR1* premutation (55–200 CGG repeats). Although initially believed to confer only the risk of transmission of FXS to offspring, it is now well-established that the premutation itself causes two specific syndromes (both with variable expression): Fragile X-associated Primary Ovarian Insufficiency (FXPOI), which is the most common known genetic cause of premature reproductive aging (Sullivan et al., 2005). Fragile X-associated Tremor/Ataxia Syndrome (FXTAS), a progressive neurodegenerative condition, manifested after the age of 50, that includes tremor and/or ataxia in most patients, and dementia, Parkinsonism, and neuropathy in a subgroup (Hagerman and Hagerman, 2013). In some but not all studies, premutation alleles have been associated with a wide range of other conditions and symptoms, including depression, anxiety, autoimmune diseases, and cognitive dysfunction, but the proportion of affected individuals and the severity of these symptoms varies from study to study (Wheeler et al., 2014).

Little is understood about the factors that lead to variability in the premutation phenotype, although X-inactivation in females is one well-established factor (Berry-Kravis et al., 2005; Hartley et al., 2012). Further, depending on the specific symptom, severity of clinical involvement is associated with the number of CGG repeats. Some symptoms, such as FXTAS, are more severe at the upper end of the premutation range, likely due to RNA toxicity (Tassone et al., 2007; Leehey et al., 2008; Hagerman and Hagerman, 2013), while other symptoms, such as FXPOI, depression, and anxiety, may be more severe in the middle of the premutation range (Ennis et al., 2006; Allen et al., 2007; Seltzer et al., 2012; Mailick et al., 2014b). This paper reports on another factor that may be relevant to the phenotypic heterogeneity of the *FMR1* premutation phenotype, namely mosaicism.

Although mosaicism in individuals carrying the full mutation of FXS has been described in the literature (Rousseau et al., 1991; Nolin et al., 1994; Tassone et al., 1999), very little is known about mosaicism in individuals diagnosed with the premutation. In one autopsy report, different CGG repeat numbers were detected in different brain regions in a premutation carrier (Lokanga et al., 2013). In other reports, although the premutation carriers had predominantly unmethylated cells, such individuals also had a small percentage of cells that were fully methylated; this is a characteristic of full mutation FXS (Allingham-Hawkins et al., 1996; Tassone et al., 1999). Recently, Pretto et al. (2014) reported premutation cases with CGG repeat size mosaicism, in which individuals had alleles of different numbers of CGG repeats either within the premutation range and/or crossing over into the full mutation range, and that such individuals exhibited more severe clinical features than those without mosaicism. Mosaicism is particularly likely at the upper premutation range. The Pretto study also suggested the possibility that mosaicism may vary by tissue type, yielding different patterns of genotype–phenotype correlations.

In the present study, DNA derived from buccal swabs was available for 100 women who previously and independently were diagnosed with the premutation. All were mothers of children with FXS. Highly sensitive assays now available (Amplidex Kit, Asuragen, Inc.) have made it possible to detect lower levels of mosaicism than previously possible and allowed classification of these premutation carriers as either non-mosaic (*n* = 41) or mosaic (*n* = 59), and to further divide the premutation mosaic group into those who had premutation mosaicism (*n* = 45) and those who had at least some detectable signal coming from cells with a mutation in the full mutation range (*n* = 14). Phenotypic data were available with respect to health, psychiatric, motor, and neurocognitive characteristics, making it possible to explore genotype–phenotype correlations associated with mosaicism. Although this is an exploratory study, based on Pretto et al. (2014) our expectation was that those with full mutation mosaicism would be more severely affected by impairments in health, psychiatric, motor, and neurocognitive characteristics than premutation carriers who were non-mosaic or were mosaic for only premutation size alleles.

## Materials and Methods

### Participants

Participants in the present study consisted of 100 premutation carrier mothers of adolescents and adults with FXS who have been followed longitudinally as part of a larger program of research involving four rounds of data collection spanning nearly a decade (Mailick et al., 2014a; Smith et al., 2016). Mothers were recruited through local media advertisements, newsletters of national disability organizations, and brochures and postings in clinics, disability listservs, and a university research registry. The participants lived in 38 United States and one Canadian province.

At the most recent round of data collection (2017), mothers ranged in age from 44 to 76 (mean = 58.4; *SD* = 7.2). The majority had at least some college education (89%) and were married (80.4%). Mothers had an average of 2.4 children (*SD* = 1.2) of whom an average of 1.8 had been diagnosed with a disability (*SD* = 0.9). Many (40.4%) had more than one child diagnosed with FXS. In families with more than one child with FXS, one was designated as the target child for the present study (i.e., the one who was most severely affected and who lived at home at the start of the research). Target children with FXS were mostly males (86%) and were in their late twenties on average (*M* = 27.7, *SD* = 7.0, ranged in age from 19 to 49 years of age). Most target children lived with their mothers (89%).

All subjects gave written informed consent in accordance with the Declaration of Helsinki. The protocol was approved by the institutional review board at the University of Wisconsin-Madison.

### Procedure

#### Genetic Data

At the start of the study in 2008, mothers provided medical records to confirm that their child had the full mutation of FXS. Mothers provided a blood sample that was sent to Kimble Genetics, Inc. to confirm their status as premutation carriers (55–200 CGGs).

In 2017, mothers provided a buccal swab in order to obtain an additional DNA sample. DNA was extracted and subjected to *FMR1* CGG genotyping (using Amplidex Kit, Asuragen) in the laboratory of Elizabeth Berry-Kravis, MD, Ph.D. This assay made it possible to better detect CGG repeat mosaicism on the expanded allele, and to divide the participants into three groups: Group 1 consisted of 45 non-mosaic premutation carriers (non-mosaic PM). Group 2 consisted of 41 premutation carriers who had mosaicism in the premutation range (PM mosaic). Group 3 consisted of 14 premutation carriers who had premutation/full mutation mosaicism (PM/FM mosaic). Group 2 mothers had at least two populations of cells with different allele sizes in the premutation range on their expanded allele. Group 3 mothers had a very small population of cells in the full mutation range on their longer allele in addition to one or multiple populations of cells with different allele sizes in the premutation range. For both Group 2 and Group 3, the predominant premutation CGG repeat number was identified. Mothers’ CGG repeats on their predominant expanded allele ranged from 67 to 138 (mean = 94.5; *SD* = 16.5). Almost all mothers (96%) had 0 AGGs on their expanded allele. Although mothers were aware of their status as premutation carriers, they were not aware of their mosaicism status.

#### Phenotypic Data

Mothers participated in telephone interviews and completed self-administered questionnaires to assess health, psychiatric, motor, and neurocognitive characteristics that have been linked in previous research to the premutation phenotype (Wheeler et al., 2017).

*Health* characteristics were measured as follows. Mothers responded to all 36 items from the SF-36 (Ware and Sherbourne, 1992), a self-report measure widely used in health outcomes and quality of life research. The SF-36 items measure eight health concepts: physical functioning (10 items, each coded 1 = limited a lot to 3 = not limited at all), bodily pain (2 items, one item coded 1 = none to 6 = very severe, and the other item coded 1 = not at all to 5 = extremely), role limitations due to physical health problems (4 items, each coded 1 = yes, 2 = no), role limitations due to personal or emotional problems (3 items, each coded 1 = yes, 2 = no), emotional well-being (5 items, each coded 1 = all of the time to 6 = none of the time), social functioning (2 items, each coded 1 = not at all to 5 = extremely), energy/fatigue (4 items, each coded 1 = all of the time to 6 = none of the time), and general health perceptions (4 items, each rated 1 = definitely true to 4 = definitely false, and one additional item rating health as 1 = excellent to 5 = poor). The SF-36 also includes a single item that provides an indication of expected change in health. The measure has excellent reliability and validity (Ware and Sherbourne, 1992).

In addition, mothers were presented with a list of 76 specific medical conditions (e.g., asthma, heart disease, sleep apnea) and they reported whether or not they had been diagnosed with each condition at any point in their lifetime (mean = 5.2, *SD* = 3.9). Mothers reported the prescription medications they were currently taking. Medications were classified as psychotropic or non-psychotropic based on the Physician’s Desk Reference Guide for Mental Health Professionals (Comer, 2002), and the number of psychotropic and the number of non-psychotropic medications were used in the present analysis. Severity of menopause symptoms was measured by items taken from the Wisconsin Longitudinal Study (Hauser et al., 1999), including hot flushes/flashes, depression, sleep disturbance, bone pain, night sweats, and other symptoms they associated with menopause, each rated as 0 = not at all, 1 = a little, 2 = somewhat, and 3 = a lot and summed. Mothers reported their height and weight, and BMI was calculated by the following formula: weight (in kilograms)/height (in meters) squared.

*Psychiatric* characteristics were measured as follows. Mothers completed the Center for Epidemiological Studies – Depression Scale (CES-D; Radloff, 1977) to measure depressive symptoms. Mothers reported the number of days in the previous week they experienced each of 20 symptoms of depression. Each symptom was rated on a scale from 0 (never*)* to 3 (5–7 days). Mothers also completed the Anxiety subscale of the Profile of Mood States (POMS; McNair et al., 1971). This subscale measures the frequency of nine anxiety symptoms experienced over the previous week, including feeling tense, shaky, or on edge, on a scale ranging from 0 (not at all) to 4 (extremely).

*Motor* characteristics included FXTAS symptoms, which were assessed by 16 items measuring tremor and shakiness, balance and walking, and other related symptoms, adapted from a questionnaire used to interview women from FXS families about problems in various clinical domains (Chonchaiya et al., 2010). In addition, mothers completed the patient questionnaire (Parts I and II) of the Movement Disorders Society modified version of the Unified Parkinson’s Disease Rating Scale (MDS-UPDRS; Goetz et al., 2007). Items measured speech, saliva and drooling, chewing and swallowing, eating, dressing, hygiene, handwriting, doing hobbies, tremor, turning in bed, getting in and out of bed or a car or a deep chair, walking and balance, freezing, sleep problems, daytime sleepiness, pain and other sensations, urinary problems, constipation, light headedness on standing, and fatigue. Each item was rated as 0 (normal) to 4 (severe).

*Neurocognitive* characteristics were measured via the Behavior Rating Inventory of Executive Function-Adult version (BRIEF-A; Roth et al., 2005), which assesses executive functioning. It consists of 75 items in nine domains, i.e., Inhibit, Shift, Emotional Control, Self-Monitor, Initiate, Working Memory, Plan/Organize, Task Monitor, and Organization of Materials. Mothers were asked to indicate the extent to which they experienced each problem in daily life on a scale from 1 (never) to 3 (often) in the past month. The reliability and validity of the BRIEF-A was well established in prior research (Roth et al., 2005, 2013).

### Background Characteristics

Mothers reported their current marital/partner status (1 = currently married or cohabiting, 0 = other), level of education (1 = some college or higher, 0 = high school graduate or less), employment status (1 = working full or part time, 0 = other), and the number of her biological children as well as the number of children with FXS.

## Analysis Plan

The analysis proceeded in three steps. First, the three groups of mothers (non-mosaic premutation, premutation mosaic, and premutation/full mutation mosaic) were compared with respect to background and genetic characteristics.

Second, using a machine learning method, the participants’ responses to each item in the interviews and questionnaires were used to predict which mosaicism group an individual belonged to. Machine learning algorithms are designed to automatically “learn” and identify patterns and structures in the data. For the present research, we used a supervised machine learning method (random forest), in which a model is built based on a “training set.” The training set is a subset of data used to fit the parameters of the model. In supervised learning, the algorithm uses the training data to automatically optimize the parameters of the model to predict group membership (i.e., non-mosaic premutation, mosaic premutation, and premutation/full mutation mosaic). The model is then applied to independent data that has not been used for training, called the “test data.” We used stratified 10-fold cross validation in order to train and test the model, where the dataset is randomly partitioned into 10 subsets of equal size and equal class proportions. The classifier is trained and tested 10 times, each holding aside a different subset for testing, with the model trained on the other nine.

From our training set, the algorithm repeatedly draws a bootstrap sample (participants drawn randomly, uniformly, with replacement) and trains a decision tree (i.e., a decision tree is derived by the algorithm based on each subset). Diversity among the set of trees is obtained not only by the different bootstrap subsets for training, but also by repeatedly drawing different subsets of the variables to consider (Breiman, 2001; Movaghar et al., 2017). The procedure yields a large number of trees that each separately predicts the classification for any new test example. The forest aggregates the prediction from all of the trees and identifies the most popular classification, as the final prediction. The most popular classification is the one that is generated most frequently from the test data. Equal contribution of the trees in classification protects the random forest classifier from “overfitting” to the training data, making it an effective prediction tool with low generalization error. The area under receiver operating characteristic curve (AUROC) was used to determine the success of the classification.

Third, the specific items that differentiated the three groups in the random forest classifier at a level of 1.00 or higher in *mean decrease in accuracy* were identified. The mean decrease in accuracy (of the random forest when a variable is omitted) shows the importance of each variable with respect to its contribution to the random forest accuracy. Mean decrease in accuracy is determined by using out-of-bag samples when a variable was randomly permuted in random forest. The out-of-bag data is the set of observations that are not used for building a particular decision tree. The more the accuracy of the random forest decreases due to the exclusion of a variable, the more important that variable is. Therefore, variables with a large mean decrease in accuracy contribute more in classification of the data (Calle and Urrea, 2010). For descriptive purposes, the three groups were compared on these specific items using analysis of covariance, with age as the covariate to adjust for the trend-level group difference in maternal age. The *F*-values are unadjusted for multiple comparisons due to the exploratory goals of this study.

## Results

### Descriptive Data

**Table 1** presents descriptive data on the three groups of premutation carrier mothers, divided by their mosaicism status. There was a trend-level difference in maternal age among the three groups. Differences were not detected between the three groups of premutation carriers with respect to marital status, level of education, employment status, number of biological children, or whether they had more than one child with FXS. Most mothers were married and nearly all had completed at least some college education. About two-thirds were currently employed. The mothers had an average of just over two biological children, and about two-fifths had more than one child with FXS.

**Table 1:** Comparison of *FMR1* premutation carriers by mosaicism status.

Variables	Non-mosaic PM (*n* = 45)	PM mosaic (*n* = 41)	PM/FM mosaic (*n* = 14)	*F*-value/ Chi-square
	Mean (SD)	Mean (SD)	Mean (SD*)*	
Age (years)	59.8 (7.2)	58.1 (7.3)	54.8 (5.8)	2.77^+^
Marital status (1 = currently married)	0.77	0.78	1.00	ns
Education (1 = some college or higher)	0.91	0.83	1.00	ns
Employment status (1 = working)	0.67	0.63	0.64	ns
Number of biological children	2.51 (1.4)	2.33 (1.1)	2.14 (0.9)	ns
Has more than 1 child with FXS	0.42	0.38	0.43	ns
CGG repeat length – long allele^a^	88.2 (13.6)	92.3 (8.7)	121.4 (16.8)	39.5^∗∗∗^
CGG repeat length – short allele	27.7 (5.1)	28.1 (7.2)	30.1 (3.6)	ns
AGG repeats (1 = zero AGG repeat)	0.93	0.98	1.00	ns

*^+^*p* < 0.10, ^∗∗∗^*p* < 0.001. ^a^For the two mosaic groups, the predominant CGG repeat on the long allele is reported.*

**FIGURE 1:**
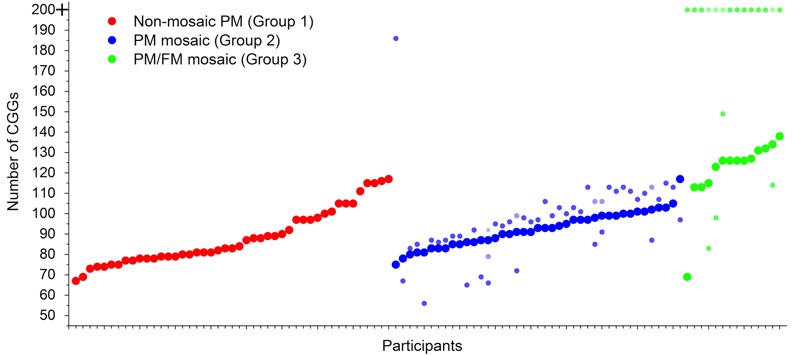
Premutation carrier mothers of children with FXS by mosaicism status (*n* = 100). The 100 participants are arrayed on the *x*-axis, each case individually. Participants are ordered by mosaicism group, first non-mosaic PM (Group 1, in red), then PM mosaic (Group 2, in blue), and finally PM/FM mosaic (Group 3, in green). Within each group, participants are ordered from lowest to highest numbers of CGG repeats. For Groups 2 and 3, large circles signify predominant premutation CGGs; small circles signify mosaicism.

**Figure 1** portrays the CGG repeat number for each of the 100 premutation carriers. For the mosaic mothers (both those who were PM mosaic and PM/FM mosaic), the predominant premutation CGG number is portrayed via larger circles, while the additional populations of cells are portrayed via smaller circles. The three groups of premutation carriers differed in their CGG repeat length on their long allele. Non-mosaic premutation carrier mothers had an average of 88.2 CGG repeats; mothers who were PM mosaic averaged 92.3 CGG repeats for the predominant allele; and the PM/FM mosaic mothers had the greatest number of CGG repeats – 121.4, on average, for the predominant allele. The groups did not differ in AGG; fewer than 10% had any AGGs.

### Classification by Machine Learning

**FIGURE 2:**
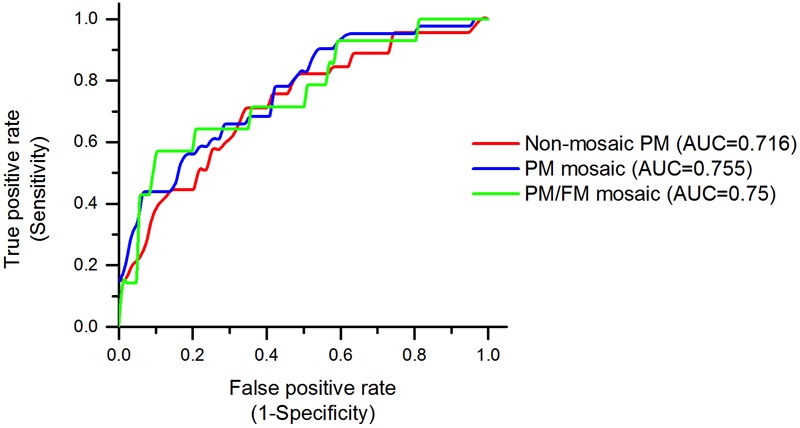
Random forest classifier’s performance, identifying mosaicism status in premutation carrier mothers. Receiver operating characteristic (ROC) curves for non-mosaic PM, PM mosaic, PM/FM mosaic have shown. ROC curves provide a comprehensive visualization to summarize the false-positive rate, or 1 – specificity versus sensitivity of the prediction method. The area under the ROC curve (AUC) illustrates how well random forest algorithm can distinguish non-mosaic PM, PM mosaic, PM/FM mosaic. The classifiers have an AUC of 0.716, 0.755, and 0.75, respectively, which are significantly higher than the baseline AUC of 0.5.

In predicting mosaicism status, we trained a random forest, which is a robust, reliable classification method with high performance and generalization power. The random forest classifier was trained based on all individual items from the following measures: SF-36, lifetime diagnoses, number of prescription medications (psychotropic and non-psychotropic), menopause symptoms, BMI, CES-Depression scale, POMS Anxiety scale, FXTAS symptoms, MDS-UPDRS, BRIEF-A. The original coding of each variable (as described above in the Measures section) was used in the machine learning classification. The classifier identified non-mosaic, PM mosaic and PM/FM mosaic cases with overall AUROC of 0.737, significantly better than random classification (i.e., AUROC of 0.5). With respect to each mosaicism group, the classifier successfully identified non-mosaic PM cases, PM mosaic and PM/FM mosaic with AUROC of 0.716, 0.755, and 0.75, respectively (**Figure 2**).

### Items Contributing to Group Classification

**FIGURE 3:**
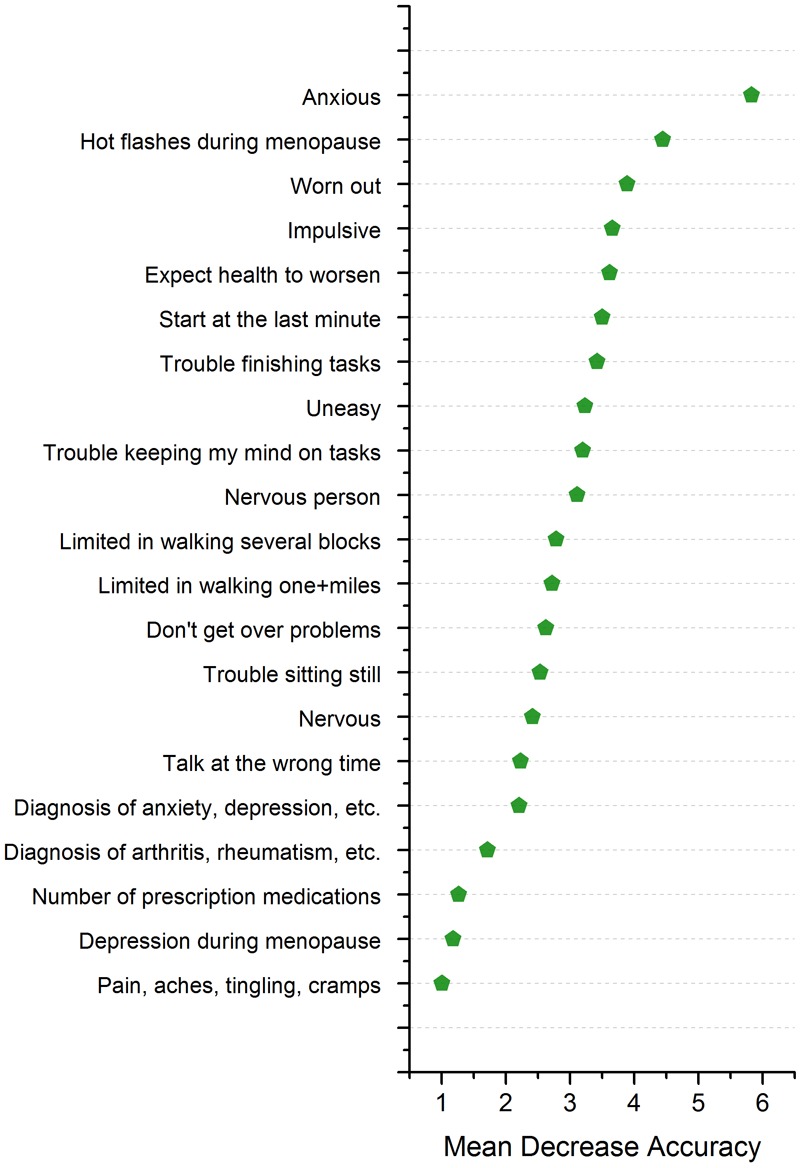
Variable importance according to mean decrease in accuracy of the random forest classifier. The mean decrease in accuracy, when a variable is permuted, shows the importance of each variable with respect to its contribution to the random forest accuracy. Variables with a large mean decrease in accuracy contribute more in classification of the data. For illustrative purposes only, variables with score higher than 1.00 are shown in the figure.

**Figure 3** presents the 21 individual symptoms or conditions that contributed the most to the classification of the three mosaicism groups, ordered by their importance as measured by the mean decrease in accuracy score (Louppe et al., 2013). **Table 2** presents the percentage of premutation carrier mothers in each mosaicism group who experienced each of these 21 symptoms or conditions. For ease of interpretation, for **Table 2** variables were recoded such that mothers were categorized as either not having a symptom or a condition (0) or having any level of severity of the symptom or condition (1). Controlling for maternal age, the three mosaicism groups were compared with respect to the percentage who experienced each of these symptoms or conditions. Significant differences (*p* < 0.05) were observed for 18 of the 21 variables, and two more differed at a trend level (*p* < 0.10). Notably, for all but two symptoms, the PM/FM mosaic mothers had the best phenotypic profile (see below).

**Table 2:** Comparison of variables with mean decrease in accuracy scores over 1.0 in classifying non-mosaic PM, mosaic PM, and mosaic PM/FM women.

Variables	Score	Non-mosaic PM (*n* = 45)	Mosaic PM (*n* = 41)	Mosaic PM/FM (*n* = 14)	*F*-value^a^
1. I felt anxious during the past week (POMS anxiety)	5.83	62.2%	80.5%	28.6%	7.09^∗∗^
2. I had hot flushes/flashes during menopause	4.44	64.3%	70.3%	16.7%	6.04^∗∗^
3. I feel worn out (SF-36)	3.89	80.0%	89.7%	85.7%	0.55
4. I am impulsive (BRIEF-A)	3.66	53.3%	53.7%	7.1%	5.74^∗∗^
5. I expect my health to get worse (SF-36)	3.62	8.9%	27.5%	14.3%	3.19^∗^
6. I start things at the last minute (BRIEF-A)	3.50	80.0%	56.1%	42.9%	5.47^∗∗^
7. I have trouble finishing tasks (BRIEF-A)	3.42	64.4%	56.1%	14.3%	6.14^∗∗^
8. I felt uneasy during the past week (POMS Anxiety)	3.24	42.2%	65.9%	35.7%	3.25^∗^
9. I had trouble keeping my mind on what I was doing (CES-D)	3.20	55.6%	63.4%	42.9%	1.10
10. I have been a nervous person (SF-36)	3.11	66.7%	90.0%	64.3%	3.89^∗^
11. My health limits me in walking several blocks (SF-36)	2.79	11.1%	35.0%	7.1%	4.92^∗∗^
12. My health limits me in walking more than a mile (SF-36)	2.72	20.0%	42.5%	7.1%	4.62^∗^
13. After having a problem, I don’t get over it easily (BRIEF-A)	2.63	62.2%	80.5%	42.9%	4.20^∗^
14. I have trouble sitting still (BRIEF-A)	2.54	64.4%	43.9%	35.7%	3.63^∗^
15. I felt nervous during the past week (POMS Anxiety)	2.42	42.2%	61.0%	21.4%	3.97^∗^
16. I talk at the wrong time (BRIEF-A)	2.23	55.6%	51.2%	14.3%	4.37^∗^
17. Lifetime diagnosis of anxiety, depression, or other emotional disorder	2.21	46.7%	43.9%	7.1%	3.94^∗^
18. Lifetime diagnosis of arthritis, rheumatism, osteoporosis, or other bone or joint disease	1.72	37.8%	26.8%	0.0%	3.93^∗^
19. Total number of prescription medications	1.27	2.28	2.07	1.21	0.59
20. I had depression during menopause	1.18	31.0%	27.0%	0.0%	2.57^+^
21. I had feelings of pain, aches, tingling or cramps during the past week (MDS-UPDRS)	1.01	77.8%	78.0%	50.0%	2.49^+^

*^+^*p* < 0.10, ^∗^*p* < 0.05, ^∗∗^*p* < 0.01. ^a^*F*-value based on ANCOVA with age as a covariate.*

*Anxiety experienced during the past week* was the symptom that contributed the most to the group classification, with only about one-quarter of the PM/FM mosaic group reporting feeling anxious during the past week as compared to twice that number in the non-mosaic PM group and over 80% of the PM mosaic group. Although not contributing as strongly to the group classification, several other items measuring anxiety during the past week reflected the same pattern (*feeling uneasy* and *feeling nervous*), with the PM/FM mosaic group having the lowest frequency of these symptoms and the PM mosaic group having the highest. PM/FM mosaic mothers were also much less likely to have received a *diagnosis of anxiety, depression, or another emotional disorder*, suggesting that the low level of anxiety symptoms experienced in the past week reflected a lower likelihood of clinically diagnosed anxiety or a related disorder. Similarly, the PM/FM mosaic mothers were less likely than the other two groups to endorse *having been a nervous person*, although it was notable that over two-thirds of each group expressed this self-assessment.

Only a few of the PM/FM mosaic mothers experienced *hot flushes or flashes during menopause*, which was the symptom that contributed second most importantly to the machine learning classification, as compared with two-thirds or more of the other two groups of mothers. Although less important in the classification, it was notable that none of the PM/FM mosaic mothers reported experiencing *depression during menopause*, whereas over 25% of the mothers in the other two groups reported this menopause symptom.

Fully one-third of the items that exceeded a mean decrease accuracy score of 1.00 (**Table 2**) reflected executive functioning problems. Mothers who were in the PM/FM mosaic group had the lowest frequency of each of these six problems: *impulsivity, starting things at the last minute, difficulty finishing tasks, having difficulty getting over a problem, trouble sitting still*, and *talking at the wrong time*.

With respect to motor functioning, again the PM/FM mosaic group appeared to be least impaired. Only one mother in that group had difficulty *walking several blocks*, whereas fully one-third of the PM mosaic mothers were limited in this activity. An even greater proportion of the mothers had difficulty *walking more than a mile*, but again only one mother in the PM/FM group had this limitation as compared with over 40% of the PM mosaic mothers. While one-half of the PM/FM group reported feelings of *pain, aches, tingling, or cramp*s during the past week, fully three-fourths of the mothers in the other two groups reported such symptoms.

None of the PM/FM mosaic mothers had a *diagnosis of arthritis, rheumatism, osteoporosis, or other bone or joint disease* at any point in their lifetime, but over one-quarter of the PM mosaic mothers reported such a diagnosis, as did more than one-third of the non-mosaic premutation carriers.

There were two items for which the PM/FM mosaic group did not have the best profile. One was the *expectation of worsening health;* the non-mosaic premutation carrier mothers were least likely to expect their health to worsen, followed by the PM/FM mosaic mothers, and the PM mosaic mothers were most likely to expect worsening health. The other was *feeling worn out*; the great majority (80% of more) of all three groups of premutation carrier mothers endorsed feeling worn out.

## Discussion

Past research has yielded differing conclusions about the premutation phenotype. Some studies reported a high prevalence of diverse symptoms and other studies raised questions about the strength of genotype–phenotype associations (Coffey et al., 2008; Hunter et al., 2009). Wheeler et al. (2014) conducted a comprehensive review of the literature and evaluated the strength of the evidence supportive of medical, reproductive, cognitive, and psychiatric features in females with the premutation. The few symptoms reported in the Wheeler et al. (2014) review as either “probably” or “definitely” related to the premutation phenotype (in the absence of FXTAS) and that were associated with CGG repeat length included neuropathy, ovarian insufficiency and fertility issues, executive dysfunction, affective disorders, and ADHD. Yet all of these symptoms were recognized to be variable in expressivity within female premutation carriers for reasons that have not yet fully been explained. The present study identified mosaicism as a possible factor contributing to this variability.

In a sample of 100 premutation carrier mothers of children with FXS, three sub-groups were identified with respect to mosaicism, based on a newly available genotyping assay. Surprisingly, the majority (59%) were found to be mosaic even though all had been previously and independently diagnosed as carrying the premutation based on a single CGG repeat number (Seltzer et al., 2012). Further, consistent with the report of Pretto et al. (2014), mosaicism was most common in the present study among those with longer CGG repeats. A sub-group of these premutation carriers (14%; those with the longest repeats) had mosaicism that included both the predominant premutation and cells with mutations that crossed over into the full mutation range (more than 200 CGG repeats).

The present study explored whether there were any clinical correlations associated with mosaicism among these female premutation carriers. Using a discovery-driven approach, a large number of self-reported symptoms was subjected to machine learning. Some of these symptoms were associated in the clinical literature with the premutation (e.g., menopause symptoms, executive dysfunction) while others were characteristic of health and mental health difficulties in the general population (e.g., heart disease, asthma, sleep apnea). The resulting algorithm differentiated the three mosaicism groups with over 70% accuracy, and the symptoms that most powerfully differentiated the groups were those previously identified as associated with the premutation. All of the symptoms identified by Wheeler et al. (2014) contributed substantially to the algorithm that differentiated the three groups: neuropathy (*pain, aches, tingling, cramps*), ovarian insufficiency and fertility issues (*hot flashes and depression during menopause*), executive dysfunction (*impulsivity, difficulty starting and finishing tasks, talking at the wrong time*), affective disorders (diagnosis of *depression and anxiety*, symptoms of *anxiety, nervousness, uneasiness*), and ADHD (*trouble sitting still*). The most prominent pattern for all of these differentiating symptoms was a substantially lower level of impairments for those who had a small population of cells with mutations in the full mutation range in addition to one or more multiple populations of cells with different allele sizes in the premutation range (the PM/FM mosaic group).

We can only speculate about the mechanism that might account for this pattern, and it must be acknowledged that the lower level of clinical impact in the PM/FM mosaic group might be due to a small group effect that warrants replication in larger studies. One possibility concerns variation in mRNA levels across the premutation range, which tends to be reduced in the upper regions of the premutation, as the gene begins to become methylated (Hagerman and Hagerman, 2013). Longer CGG repeat lengths have been associated with a more severe phenotype for some symptoms (particularly FXTAS symptoms; Hagerman and Hagerman, 2013). Yet for other symptoms a curvilinear association with CGG repeat length has been reported (menopause symptoms, depression, anxiety; Roberts et al., 2009; Seltzer et al., 2012), with less severe symptoms in the lower and upper ranges of the premutation. Although the most striking finding of the present comparison was the lower level of clinical affectedness of those with PM/FM mosaicism (Group 3), it was also the case that for about half of the symptoms that contributed most powerfully to the algorithm, those with premutation-only mosaicism (Group 2) had the worst symptoms. In the present study, those with premutation mosaicism were in the mid-range of the premutation distribution (averaging 92 CGG repeats, as compared with 121 repeats for the PM/FM mosaic mothers). Thus, another possible reason why those with PM/FM mosaicism might have been the least clinically affected was because the symptoms that emerged in the algorithm had a curvilinear association with CGG repeat length, as has been reported in earlier studies (Ennis et al., 2006; Allen et al., 2007; Roberts et al., 2009; Seltzer et al., 2012; Mailick et al., 2014b). We cannot determine from the present data whether the patterns we report are due to mosaicism *per se*, or to the CGG repeat length itself. Both could be biomarkers of a shared underlying process, but larger numbers of premutation carriers are needed to separate these effects. Further, whereas our results are strongly suggestive of novel associations, there could be additional associations yet to be uncovered with larger sample sizes. It is possible that the symptoms and behaviors reported in this study may have been due to other mutations, but we did not carry out a genetic analysis that would have revealed such mutations. Replicate investigations will be critical to moving the state of the knowledge forward.

The three groups did not differ in most background characteristics (education, marital status, employment status, number of biological children, and their likelihood of having more than one child with FXS). However, although not a significant difference, the three groups differed in age at a trend level. Therefore, age was statistically controlled in the group comparisons, and with age controlled the mosaicism group differences remained significant. Yet age effects warrant additional investigation in future research as these remain an alternative explanation for the present findings.

An additional important goal for future research concerns the approach to phenotyping, as the present data were based on self-reports. Although many of the measures we used have been validated in past research, and report of symptoms seemed consistent across measures based on comparable symptoms from different measures contributing to group classification, direct clinical confirmation of the symptoms that contributed to the algorithm would strengthen conclusions about genotype–phenotype correlations. The lack of data about methylation (activation ratios) and mRNA level are other limitations of the present study. It also must be noted that these analyses were conducted on DNA from buccal tissue and it is unclear if mosaicism is the same across tissues or if a different result would be seen if correlations were done with DNA derived from blood samples. Further work will be required to clarify this question.

Juxtaposed against these limitations are some strengths of the present research including the combination of genetics, machine learning, and psychosocial self-report measures, and the relatively large proportion of premutation carriers who were mosaic. If the patterns reported here are confirmed in future research, then the currently available more sensitive diagnostic assays will make it possible to identify sub-groups at differential clinical risk based on an additional premutation biomarker, namely mosaicism.

## Author Contributions

MM: designed the research and wrote the manuscript. AM and JH: statistical analysis and reviewed the manuscript. JG, LD, PR, MeB, and MuB: initial input on study design and reviewed the manuscript. LZ: coordinated genetic assays and reviewed the manuscript. JJ: conducted genetic assays and reviewed the manuscript. DP: supervised statistical analysis and reviewed the manuscript. EB-K: initial input on study design, supervised genetic assays, and reviewed the manuscript.

## Conflict of Interest Statement

MM: Chair of the Scientific Review Board, John Merck Fund Developmental Disabilities Program. EB-K has received funding from Seaside Therapeutics, Novartis, Roche, Alcobra, Neuren, Cydan, Fulcrum, Neurotrope, BioMarin, GW, Marinus, Zynerba and Ovid Pharmaceuticals to consult on trial design or development strategies and/or conduct clinical trials in FXS, Rett syndrome, CLN2 or Down syndrome, from Vtesse/Sucampo to conduct clinical trials in NP-C, and from Asuragen Inc. to develop testing standards and resources for FMR1 testing. The other authors declare that the research was conducted in the absence of any commercial or financial relationships that could be construed as a potential conflict of interest.
